# *In Vivo* Antimalarial Evaluation of Crude Extract, Solvent Fractions, and TLC-Isolated Compounds from *Olea europaea* Linn subsp. *cuspidata* (Oleaceae)

**DOI:** 10.1155/2020/6731485

**Published:** 2020-05-13

**Authors:** Gebretekle Gebremichael Hailesilase, Yarra Rajeshwar, Gebremedhin Solomon Hailu, Gereziher Gebremedhin Sibhat, Helen Bitew

**Affiliations:** ^1^Department of Pharmacy, Adigrat University, Adigrat, Ethiopia; ^2^Department of Medicinal Chemistry, Mekelle University, Mekelle, Ethiopia; ^3^Department of Pharmacognosy, Mekelle University, Mekelle, Ethiopia

## Abstract

Malaria is a major global public health problem caused by *Plasmodium* parasites. Drug resistance is becoming a great challenge. New drugs with novel mechanism of action are urgently required. In malarious countries, medicinal plants are commonly used for malaria treatment. *Olea europaea* is traditionally used against malaria in Ethiopia. The aim of this study was to isolate and evaluate antimalarial activity of chemical constituents extracted from *Olea europaea* against chloroquine-sensitive *Plasmodium berghei*-infected mice. Stem bark of *Olea europaea* was extracted with 80% methanol and fractionated with three solvents. The butanol fraction was subjected to isolation with preparative thin-layer chromatography (PTLC). Acute oral toxicity studies were conducted in mice as per the Organization for Economic Co-operation and Development (OECD) guideline 425. Antimalarial activities of the test substances were evaluated using Peter's 4-day suppressive test. The crude extract showed significant (*p* < 0.01) antiplasmodial activity at all doses with a chemosuppression value of 52.40% at a dose of 600 mg/kg. All fractions also suppressed parasitaemia significantly (*p* < 0.05), the highest suppression (45.42%) being with butanol fraction. In the phytochemical analysis, two compounds were isolated. Both compounds showed significant (*p* < 0.05) antimalarial activities. Compound C inhibited parasitaemia up to 38.19% at a dose of 200 mg/kg. The crude extract, butanol fraction, and isolated compounds also prolonged survival time of mice. No sign of toxicity and mortality was seen in the test substances at up to a single dose of 2 g/kg. Findings of the current study may confirm the traditional antimalarial claim of *Olea europaea* and its relative safety as well as the potentiality of compound C for further investigations.

## 1. Introduction

Malaria is a deadly disease caused by *Plasmodium* parasites transmitted to human via bites of *Plasmodium* infected female *Anopheles* mosquito. *P. falciparum*, *P. vivax*, *P. malariae*, *P. ovale*, and *P. knowlesi* are *Plasmodia* species that cause human malaria. *P. falciparum* is the most virulent malaria parasite in Africa and accounts for most malarial deaths globally [1, 2]. Malaria is a major public health problem globally. An estimated 219 million cases and 435, 000 deaths of malaria occurred worldwide in 2017. About 92% of all cases and 93% of all deaths of malaria occurred in Africa in 2017. More than two thirds of all malaria deaths occur in children under five years of age globally [3]. In Ethiopia, *P. falciparum* and *P. vivax* account for about 66% and 34% of all malaria cases, respectively [1]. The disease remains a great health problem in the country. There were about 621,345 malarial cases and 1561 deaths in 2015 in Ethiopia [4]. About 6% of global malaria cases and 12% of global cases and deaths of *P. vivax* occur in Ethiopia. More than 75% of global malarial deaths occur in four countries: Ethiopia, Indonesia, India, and Pakistan [3].

The emergence of *Plasmodium* and mosquito resistance to antimalarial drugs and insecticides, respectively, may trigger rise in global mortality from malaria. In some parts of Asia, *P. falciparum* became resistant to most antimalarial drugs. Between 2010 and 2013, 53 countries reported mosquito resistance to at least one insecticide [5]. Resistance of *P. falciparum* to quinine is high in Asia and South America [6]. Chloroquine-resistant *P. falciparum* spread to almost all malarious countries. Chloroquine-resistant parasites in Africa emerged due to mass drug administration and intrinsic entomological factors [7]. On the other hand, artemisinin-resistant *Plasmodium* species emerged due to frequent use of oral artemisinin-based monotherapies. Hence, the WHO discourages such regimens and promotes artemisinin combination therapy [5].

Searching new antimalarial agents from nature with novel mechanism of action is, therefore, a rational approach to overcome the burden of the disease associated with drug resistance [8]. About 20% of malaria patients use herbal remedies for malaria treatment in malarious areas [9]. The most useful antimalarial drugs, quinolones and artemisinins, were discovered from plants. Caventou and Pelletier isolated quinine in 1820 from Cinchona. Quinine served as scaffold to synthesize other antimalarial drugs [10]. On the other hand, artemisinin was discovered from *Artemisia annua*, a popular antimalarial plant [11].

The olive tree (Oleaceae family) consists of 600 species divided over 30 genera. The genus *Olea* is member of this family comprising 30 species. *Olea europaea* subsp. *cuspidata* belongs to the genus *Olea.* It is widespread from South to East Africa and from Southeast Asia to Southwest China and Arabia. It is an evergreen tree or shrub reaching 15 m in height. It has grey to brownish-blackish, smooth-to-rough bark [12]. Locally*, O. europaea* Linn subsp. *cuspidata* is known as *Weira* in Amharic [13] and *Awli-e* in Tigrigna [14]. It is used for various ethnomedicinal purposes in Ethiopia. Different parts of the plant are used to fumigate pots of local alcoholic drinks. Twigs are popularly used for keeping teeth hygiene [13]. Its bark is used to treat tapeworm, ascariasis, and diarrhea [15]. In Kilte Awulaelo district, Tigray, Ethiopia, stem bark of *O. europaea* (Linn subsp. Cuspidata) is traditionally used to treat malaria [14]. The Kenyan people also use the plant for similar purpose [16].

The plant is investigated to have antifungal [17], antibacterial [18], anti-inflammatory [19], antioxidant [20], and antihypertensive activities [21]. In addition, phytochemistry investigations led to the isolation of different compounds from *O. europaea* bark including lignan glycosides and the coumarins esculetin, scopoletin, oleuropein, and scopolin [15, 22]. The leaf and fruit are rich sources of oleuropein, the most abundant phenolic compound present in olive fruit [23]. However, no *in vivo* study so far has been reported confirming the traditional antimalarial claim of the bark of *O. europaea*. The objective of the present study was, therefore, to investigate the antimalarial activity of crude extract, fractions, and isolated compounds from *O. europaea* on *P. berghei* infected mice. The study was also done to evaluate the safety and effects of crude extract, fractions, and isolated compounds from *O. europaea* on body temperature, body weight, and mean survival time of *P. berghei* infected mice.

## 2. Materials and Methods

### 2.1. Plant Material

The stem bark of *O. europaea* was collected from a forest found in Tigray, Ethiopia. The plant was identified and authenticated by a taxonomist at the Department of Biology, University of Gondar (Ethiopia). A voucher specimen was deposited there with a voucher number of GT0025/2010 for future reference.

### 2.2. Experimental Animals, Test Parasite, and Standard Drug

Both sexes of Swiss albino mice aged 7-8 weeks and weighing 23–35 g were obtained from the Department of Pharmacology, College of Health Sciences, Mekelle University. Mice were kept for acclimatization for one week prior to experiment and they were exposed to 12 hours of light and 12 hours of dark. They were given water and standard pellet diet. Chloroquine sensitive strain of *Plasmodium berghei* (ANKA strain) was obtained from the Ethiopian Public Health Institution (EPHI). On weekly basis, the parasite was kept by serial passage of blood from infected to noninfected mice. At the end of each experiment, the mice were euthanized with halothane. Chloroquine phosphate was obtained from Addis Pharmaceutical Factory (APF), Adigrat, Tigray, Ethiopia.

### 2.3. Extraction and Fractionation

The collected stem bark of *O. europaea* was cleaned, dried under shade at room temperature, and ground to powder using a mechanical grinder. One kg of the powder was soaked with 5 L of 80% methanol and kept on an orbital shaker (Bibby Sterilin, UK) at 130 rotations per minute at room temperature for three days. The extract was filtered using muslin cloth and then Whatman filter paper number 1 (GE healthcare company, India). The marc was reextracted two times by adding fresh 80% methanol. The filtrate was dried in a drying oven (Genlab, England) at a temperature not exceeding 40°C. The crude extract was stored in a refrigerator at 4°C for further investigations.

The crude extract was further fractionated with three solvents of different polarity, that is, distilled water, n-butanol, or simply butanol (Carlo Erba, France), and chloroform (Carlo Erba, France). Twenty grams of the crude extract was first dissolved in 200 ml of distilled water. Then, 200 ml of butanol was added to the mixture and the suspension formed was shaken in a separatory funnel. The suspension was shaken three times subsequently by adding the same quantity of fresh butanol to obtain the butanol fraction. The aqueous residue was then similarly shaken with 200 ml of chloroform three times and the chloroform fraction was collected. The butanol, chloroform, and aqueous fractions were dried and stored in the same way as the crude extract. The fractionation procedure was repeated twice.

### 2.4. Isolation

The butanol fraction was subjected to isolation using preparative thin layer chromatography (PTLC). The PTLC plates were prepared (coated) using silica gel GF 254 (Loba Chemie, India), automatic TLC plate coater (Camag, Switzerland), distilled water, and 20 × 20 cm TLC glass plates (Camag, Switzerland). Chloroform: n-hexane: methanol (4 : 1 : 0.25) was used as mobile phase. The developed chromatograph was viewed under UV lamp (Uvitec, UK) at wavelengths of 254 and 365 nm [24]. The chromatographic zone was coded as A, B, C, and D based on the ascending order of retention factor (RF) values of bands. Each band was carefully marked with pencil, scrapped, collected, and dissolved in chloroform and methanol (1 : 1). The resulting mixture was filtered and the solvent was evaporated using a drying oven [24, 25]. Finally, purity of the isolated compounds was monitored using analytical TLC plate (Merck, Germany) by viewing under UV lamp at the specified wavelengths. Mixed or contaminated compounds were repurified.

### 2.5. Biological Activity Tests

#### 2.5.1. Acute Oral Toxicity Test

Oral toxicity studies were performed as per the Organization for Economic Co-operation and Development (OECD) guideline 425 [26], where the limit test dose of 2 g/kg body weight was used. The tests were first done on the crude extract and then on the solvent fractions and isolated compounds. Five noninfected female Swiss albino mice aged 8–12 weeks and weighing 23–30 g were used for each test. The mice were fasted for three hours before dosing and one hour after dosing, but they were not deprived from water. Fasted weight of mice was used for dose determination. In each test, one mouse was dosed first and then followed up. The remaining four mice were then dosed after the survival of the first mouse. Each mouse received an oral single dose of 2 g/kg body weight of the test substances dissolved in distilled water in a total volume of 10 ml/kg body weight. The mice were observed continuously for the first 30 minutes for any signs of toxicity, followed by observations every four hours for the first 24 hours and once a day for the next 13 days. Gross physical and behavioral changes like hair erection, loss of appetite, diarrhea, sleep, and coma were observed [26].

#### 2.5.2. Antimalarial Activity Test

The experiment was established using randomized design dividing male Swiss albino mice into five groups of six mice per experiment. Antimalarial activity was evaluated by the standard Peter's 4-day suppressive test in all mice infected with *P. berghei* [27]. Albino mice previously infected with *P. berghei* served as donors. Parasitaemia level of the donor mice was 20–30%. These mice were sacrificed by head hammering and the blood was collected in a Petri dish having 0.5% trisodium citrate (an anticoagulant) by severing jugular vein. The blood was then diluted with physiological saline (0.9%) to prepare 1 ml of blood having 5 × 10^7^ infected erythrocytes. Each mouse was injected intraperitoneally with 0.2 ml of the blood suspension containing 1 × 10^7^ parasitized erythrocytes [28, 29].

Treatment began three hours after the infection on day 0 and continued daily for four days (from day 0 to day 3). Mice in the treatment groups (groups I, II, and III) received the crude extract at doses of 200, 400, and 600 mg/kg body weight, respectively. Group IV (negative control) received 10 ml/kg body weight of vehicle (distilled water), while group V (positive control) was given chloroquine phosphate at a dose of 25 mg/kg body weight [27, 30]. All doses were dissolved in the vehicle and given in a volume of 10 ml/kg body weight with oral gavage [26]. The same procedure was used for the fractions and isolated compounds with the exception of reduction of group (for the isolated compounds only) and doses (Table 1).

On day 4, blood sample was taken from tail snip of each mouse. Thin smear was then prepared on a frosted microscope slide, fixed with methanol, and stained with 10% Giemsa solution (BDH Chemicals Ltd, England) for 20 minutes. Two smears were prepared for each mouse. The slides were examined under the microscope with an oil immersion objective of 100x magnification power to determine percent parasitaemia and suppression by counting a minimum of three fields per slide. Smears were codified and read by both the investigators and laboratory technicians to minimize the probabilities of biases and errors. Inhibition of parasite growth was evaluated by comparing with the nontreated groups. Percent parasitaemia and suppression were calculated as follows [30, 31]:

(1) $$\begin{array}{c} \%\text{ parasitaemia}=\left(\frac{\text{number of parasitized red blood cells}}{\text{total red blood cells counted}}\right)\times 100,\\ \%\text{ suppression}=\left(\frac{\text{parasitaemia in negative control}-\text{parasitaemia in the treated group}}{\text{parasitaemia in negative control }}\right)\;\times 100. \end{array}$$

#### 2.5.3. Determination of Mean Survival Time, Body Weight, and Temperature

Mean survival time (MST), body weight, and temperature of the infected mice were also used as additional parameters to evaluate antimalarial activity under the 4-day suppressive test. Rectal temperature of the mice was measured with rectal thermometer, just before infection and then daily up to day 4. Temperature taken on D_0_ and D_4_ was statistically analyzed. On the other hand, body weight of each mouse was measured on day 0 and on day 4. The number of days each mouse survived after infection was recorded. Mean body weight and MST of mice in each group was determined as follows [32, 33]:

(2) $$\begin{array}{c} \text{MST}=\frac{\text{total survival time }\left(\text{days}\right)\text{ of all mice in a group}}{\text{total number of mice in that group}},\\ \text{mean body weight}=\frac{\text{total body weight of all mice in a group}}{\text{total number of mice in that group}}. \end{array}$$

### 2.6. Statistical Analysis

Data were analyzed using computer software SPSS version 21. One way analysis of variance (ANOVA) followed by post-hoc Tukey test was employed to compare parameters among groups. Two-tailed paired *t*-test was applied to compare body temperature and weight before and after treatment within group. Results were expressed as mean ± standard error of the mean (mean ± SEM). All data were analyzed at 95% confidence interval, and results were considered statistically significant at *p* value <0.05.

### 2.7. Ethical Statement

Ethical approval of this study was given by Health Research Ethics Review Committee (HRERC) of College of Health Sciences, Mekelle University, Ethiopia. Accordingly, the experimental animals were used and sacrificed in a humane manner.

## 3. Results

### 3.1. Yield of the Crude Extract and Solvent Fractions

The maceration of stem bark of *O. europaea* using 80% methanol resulted in 22.19% of yield. The dried and powdered form of the crude extract had a brown color. On the other hand, the butanol fraction gave the highest percentage of yield among the three fractions while chloroform fraction gave the least yield (Table 2).

### 3.2. Isolation

**Figure 1:**
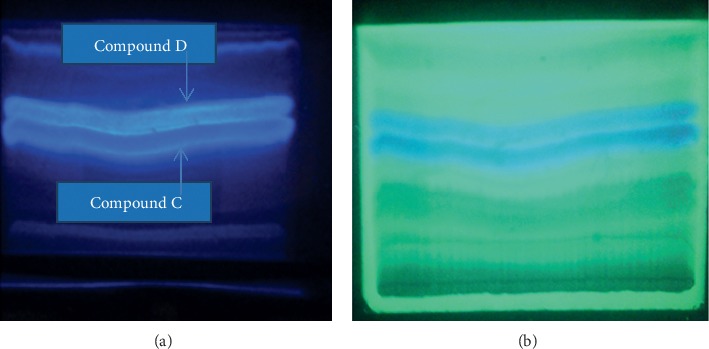
PTLC chromatogram of compounds C and D in the first step of isolation at wavelengths of 365_nm_ (a) and 254_nm_ (b).

**Figure 2:**
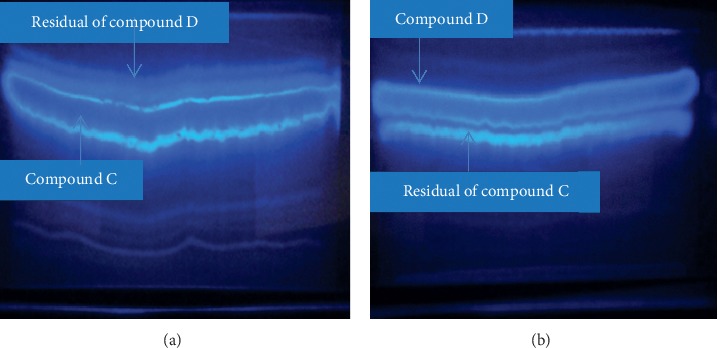
PTLC chromatogram of compounds C and D in the first step of purification at a wavelength of 365 nm.

**Figure 3:**
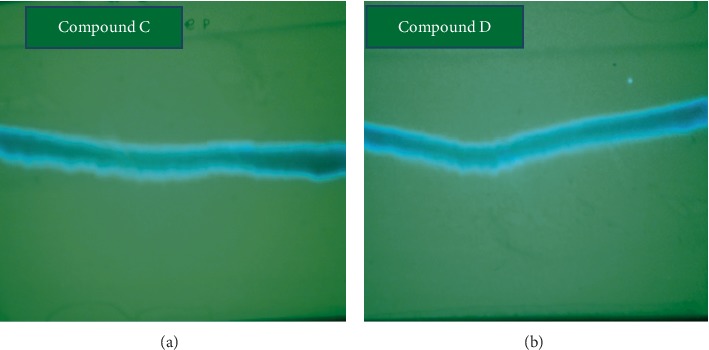
TLC chromatogram of the finally purified form of compounds C and D at a wavelength of 365 nm.

In the current analysis of *O. europaea* by PTLC, two compounds, C and D, were isolated. Compound C appeared in the chromatographic zone as a bright yellow in day light, dark blue under UV 254_nm_, and bright blue under UV 365_nm_ (chloroform: n-hexane: methanol (76 : 19 : 5)). It was isolated as a dark green powder with RF value of 0.56. Compound D was also isolated as a yellow powder having RF value of 0.61 (Figures 1–3). The amount of the compounds obtained at the first step of isolation was 592 and 625 mg for compounds C and D, respectively. From these, a quantity of 336.3 mg of compound C (56.8% of yield) and 384.38 mg of compound D (61.5% of yield) was collected at the final step of purification.

### 3.3. Acute Oral Toxicity Test

In the acute toxicity study of crude extract, fractions, and isolated compounds of the plant, there were no gross physical and behavioral changes such as hair erection, diarrhea, sleepiness, coma, and loss of appetite. Besides, no death occurred during the follow-up period of 14 days when an oral single dose of up to 2 g/kg was used, which is 10 times the minimum effective dose tested for the crude extract and butanol fraction (200 mg/kg) and 20 times for compound C (100 mg/kg).

### 3.4. Antimalarial Activity of the Crude Extract

All doses of the crude extracts of *O. europaea* showed significant parasitaemia inhibition (*p* < 0.001) in a dose-dependent manner in mice infected with *P.berghei* compared to the negative control group, but none of the extract doses completely cleared the parasite. Mice treated with the standard drug (chloroquine phosphate) at a dose of 25 mg/kg were completely parasite-free on day 4. The dose 600 mg/kg showed the highest suppression (52.40%) among all the tested doses. Comparison within three doses of the extract showed that statistically significant difference (*p* < 0.05) in parasitaemia reduction was noticed only between the higher and lower doses (Table 3). Mice treated with 400 and 600 mg/kg doses of the extract had significantly longer MST (*p* < 0.05) than mice in the negative control. However, the dose of 200 mg/kg did not have a significant effect compared to that of the negative controls (*p* > 0.05). In addition, there was no significant difference in MST between groups treated with 600 and 400 mg/kg although the extract displayed a dose-dependent effect. All mice treated with the standard drug survived up to 28 days (Table 4).

As summarized in Table 4, all doses of the extract prevented weight loss of the experimental mice due to parasitaemia significantly (*p* < 0.05) compared to the negative control. No significant loss of body weight was also noticed between day 0 and 4 in mice treated with higher and middle doses of the extract. Nevertheless, no statistically significant difference was observed among the doses. Moreover, mice in the lower dose and the negative control showed significant difference in body weight on day 4 as compared with their own weight on day 0. The standard drug prevented weight loss significantly (*p* < 0.05) compared to all treatment groups. Besides, no significant reduction of body weight was observed between day 0 and 4 in this group.

**Figure 4:**
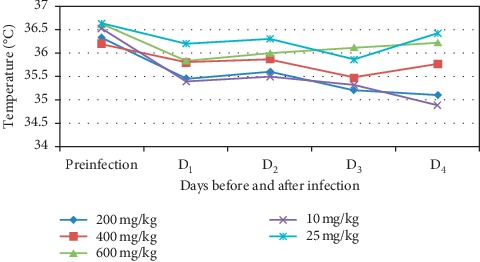
Effect of the crude extracts of *O. europaea* on rectal temperature of *P. berghei* infected mice. Preinfection = just before infection; D_1_ = day one; D_2_ = day two; D_3_ = day three; D_4_ = day four after infection.

The extract also produced a dose-dependent effect on preventing drop in body temperature on day 4 after the infection. The upper doses and standard drug showed again significant difference (*p* < 0.05) compared to the negative control on the parameter while no statistically significant difference was observed among treatment doses. On the other hand, comparison of body temperature within groups on day 0 and 4 indicated a significant (*p* < 0.05) drop of temperature in both treatment and negative control groups (Figure 4).

### 3.5. Antimalarial Activity of the Solvent Fractions

All doses of the solvent fractions of *O. europaea* reduced parasitaemia level at different degrees compared to the negative controls in a dose-dependent manner (Table 5). Mice treated with all doses of butanol fraction had significantly reduced parasitaemia level (*p* < 0.001) compared to the negative control group. The dose 400 mg/kg showed the highest suppression (45.42%) among all doses of the fractions. In addition, this was the only dose that significantly prolonged the MST in the experimental mice compared to the negative control mice and its lower dose (*p* < 0.01). Comparing between all doses of butanol fraction, a statistically significant difference in parasite inhibition was noticed only between 400 and 100 mg/kg (*p* < 0.01). The standard drug again cleared the parasite from the mice and no death occurred in these mice during up to 28-day follow up.

The chloroform fraction also showed significant chemosuppression in all doses compared to the negative control (*p* < 0.05), the highest suppression being with 400 mg/kg. Nevertheless, there was no significant difference in parasitaemia suppression among doses of the fraction. Similarly, the three doses did not show significant difference with regard to the MST of experimental mice compared to that of the negative control. Compared to the negative control group, significant parasitaemia reduction (*p* < 0.05) was observed in the higher doses of aqueous fraction, but none of the doses showed a significant effect on the MST of the experimental mice. Moreover, lower dose of this fraction was the only dose with no significant effect (*p* > 0.05) on parasitaemia from all doses of the three fractions (Table 5).

The three fractions exhibited various degrees of preventing body weight loss of experimental mice (Table 6), but only the higher dose of butanol fraction displayed a statistically significant effect compared to that of the negative control (*p* < 0.05). Besides, the difference was not statistically significant between doses of the fractions. The standard drug showed a significant effect compared to the negative control. Comparison made on day 0 and 4 within group showed that mice treated with the higher and middle doses of butanol and aqueous fractions did not show significant difference in their body weight (*p* > 0.05).

**Figure 5:**
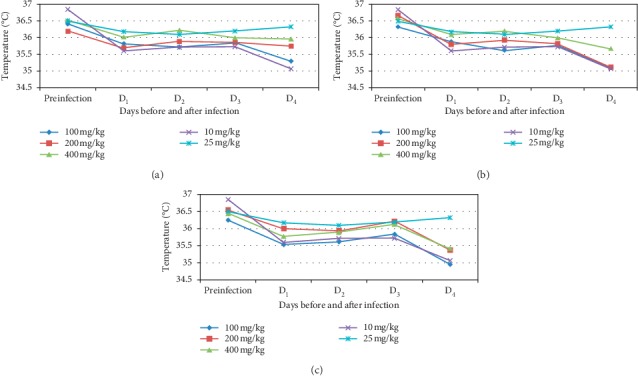
Effect of butanol (a), chloroform (b), and aqueous (c) fractions of *O. europaea* on rectal temperature of *P. berghei* infected mice. Preinfection = just before infection; D_1_ = day one; D_2_ = day two; D_3_ = day three; D_4_ = day four after infection.

The higher and middle doses of butanol fraction showed a significant effect (*p* < 0.01) on preventing body temperature drop compared to the negative control group on day 4 after infection (Figure 5). However, comparison between doses of this fraction did not show statistically significant differences. Besides, the aqueous and chloroform fractions failed to show any significant effect on body temperature (*p* > 0.05). Similarly, mice in all groups, except in the upper doses of butanol fraction and standard drug, had a significantly reduced temperature on day 4 in comparison with their own body temperature on day 0.

### 3.6. Antimalarial Activity of the Isolated Compounds

The isolated compounds exhibited a dose-dependent chemosuppressive effects ranging from 14.79 to 38.19% (Table 7). Both doses of compound C significantly reduced parasitaemia level (*p* < 0.001) of mice compared to the negative control group. Upper dose of compound C produced the highest (38.19%) suppression compared to all doses of the isolated compounds. Moreover, mice which received the upper dose of the compound had longer MST (*p* < 0.05) than the mice in the negative control. Compound D also reduced parasitaemia level and prolonged MST significantly (*p* < 0.05) at its higher dose, but the compound showed inferior activity compared to compound C. Its lower dose did not even produce any significant effect on parasitaemia and MST. On the other hand, neither of the isolated compounds showed suppressive effects comparable with that of the standard drug.

Compound C exhibited statistically significant difference (*p* < 0.05) at a dose of 200 mg/kg in terms of preventing loss of body weight compared to that of the negative control (Table 8). The difference, however, was not significant compared to its lower dose. Compound D did not show a significant difference compared to the negative control (*p* > 0.05) on the parameter. Mice in the negative control and treatment groups showed statistically significant (*p* < 0.05) weight loss on day 4 compared with their own weights on day 0. In comparison to the negative control and lower doses of the isolated compounds, the standard drug showed a significant effect on the parameter. In addition, no significant weight change was observed in the standard drug treated mice on day 4 compared with their weight on day 0.

**Figure 6:**
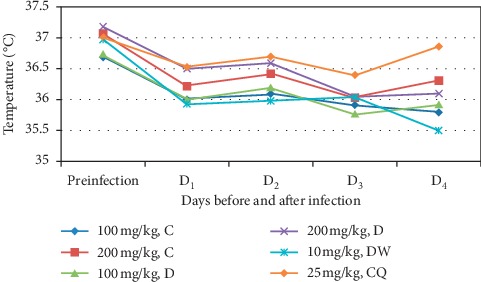
Effect of compounds C and D on rectal temperature of *P. berghei* infected mice. Preinfection = just before parasite inoculation; D_1_ = day one; D_2_ = day two; D_3_ = day three; D_4_ = day four after infection; DW = distilled water, CQ = chloroquine phosphate; C = compound C; D = compound D.

Although the isolated compounds had improved body temperature of the infected mice on day 4 in comparison with negative control (as depicted in Figure 6), neither of them produced statistically significant effect (*p* > 0.05). As compared with their own body temperature, there was also significant reduction between day 0 and day 4 (*p* < 0.05) in all groups. However, no significant change was observed in body temperature in mice which received the standard drug and higher dose of compound C on day 4 as compared with their own body temperature on day 0. The standard drug still had a significant effect on preventing drop in body temperature compared to the negative control and compound D.

## 4. Discussion

There are lots of solvents used for extraction of herbal remedies. However, extraction by hydroalcohol, notably hydromethanol, gives a higher number and more types of chemical constituents than many other solvents [34]. The plant in this study was extracted by maceration with hydromethanol. The crude extract was fractionated with three solvents to assess the nature and concentration of the metabolites responsible for antimalarial activity. The butanol fraction gave the highest yield (69.75%). The presence of high concentration of the chemical constituents in the plant with better solubility in butanol may be the reason for the difference.

Results of the acute oral toxicity test revealed that the crude extract of *O. europaea*, solvent fractions, and isolated compounds showed no gross physical and behavioral changes as well as mortalities till the end of follow-up of 14 days using a dose of up to 2 g/kg, which is 10 times the minimum effective dose tested for the crude extract and butanol fraction and 20 times for compound C. A test substance is considered a good candidate for further studies if its lethal dose (LD_50_) is three times higher than the minimum effective dose [31], suggesting that the crude extract, butanol fraction, and compound C can be good candidates for further studies.

In the present study, the crude extract, solvent fractions, and isolated compounds were investigated for their antiplasmodial activity using Peter's 4-day suppressive test to evaluate blood schizonticidal activity during early infection [27]. The crude extract showed a parasitaemia inhibition of 31.63%, 44.55%, and 52.40% at 200 mg/kg, 400 mg/kg, and 600 mg/kg, respectively. This chemosuppression obtained during the 4-day early infection test indicates the schizonticidal activity of the extract. The longest MST of *P. berghei* infected mice was seen with the highest parasitaemia suppression. This is similar to the previous study in which mice with maximum parasitaemia inhibition had the longest MST [35]. This may suggest that the extract in the current study had suppressed and reduced the overall pathologic burdens of the parasite on the study mice.

Mice treated with the standard drug (chloroquine phosphate) were free of the parasite on day 4 as reported in the previous studies [25, 36]. The standard drug treated mice in this study had also microscopically undetectable parasitaemia level on day 4 in all the experiments. This was supported clinically since all mice survived 28 days looking healthy. This may give evidence that the parasite used in this study is sensitive to chloroquine and the 4-day suppressive test using this rodent malaria model remains reliable in the evaluation of antimalarial substances.

Body weight of mice was another parameter used in this study as it is one important character of malaria infection in rodents [32]. The extract protected weight loss of the study mice as compared to negative control. The protection may have been achieved via parasitaemia inhibition or appetite improvement. Malaria in rodents is associated with a drop of body temperature rather than fever. Deleterious effect of the infection on heat production and conservation, increased serotonin in the brain of rodents, and decreased metabolic rates are the hypothesized mechanisms [33]. The extracts prevented temperature fall compared to the negative control. This might be due to hyperthermic effect of the extract on mice via adjustment of pathological processes and metabolic rates.

The observed chemosuppressive effects of the extract coupled with its prevention of body weight and temperature reduction as well as improvement of MST of mice indicates the protective and the antiplasmodial activity of the plant. Using Peter's 4-day suppressive test, a compound reducing parasitaemia level by ≥30% is considered active [31]. Therefore, the crude extract from this plant is considered active against the tested plasmodia at the tested doses. This in turn may justify the traditional antimalarial claim of the plant by the peoples of Tigray [14] and Kenya [16]. Previous *in vitro* antimalarial study on leaf extract of *O. europaea* (differing in subspecies) reported that the plant was found to be almost inactive [37]. This dissimilarity could be due to variations in subspecies and the test models, that is, *in vitro* versus *in vivo.*

*O. europaea* possesses several secondary metabolites such as diterpenes, triterpenes, sesquiterpenes, coumarins, lignans, flavonoids, and phenolic compounds [18, 22]. These metabolites have been reported to demonstrate antimalarial activities in other medicinal plants [8]. The antiplasmodial activity of the plant observed in this study may, therefore, be due to the presence of these metabolites, acting either in combination or individually. Oleuropein is a phenolic compound abundant in *O. europaea.* It has a protective effect on red blood cells from oxidative damage [22]. Compounds with antioxidant activity in turn affect plasmodia parasites via inhibition of haem polymerization, as the unpolymerized haem is toxic to the parasite [38]. Therefore, the phenolic compound may also contribute to the noticed antiplasmodial activity of this plant.

Similarly, the three fractions produced a dose-dependent parasitaemia inhibition. The butanol fraction showed the relatively highest parasitaemia reduction followed by chloroform fraction. All doses of butanol fraction exhibited statistically significant difference as compared to negative control, the highest suppression being with 400 mg/kg (45.42%). Among the fractions, only the higher dose of butanol fraction prolonged the MST compared to the negative control. The chloroform and aqueous fractions also displayed significant chemosuppressive activities in a dose-dependent manner. Nonetheless, their chemosuppressive activity was relatively lower compared to the butanol fraction. The difference could be due to the lower concentration of the secondary metabolites or the lower number of the antimalarial secondary metabolites in chloroform and aqueous fractions. The same order of chemosuppressive effects of butanol, chloroform, and aqueous fractions was reported from the root extract of *D. angustifolia* [30] and the seed extract of *D. angustifolia* [39]. In the current study, mice treated with chloroform and aqueous fractions had also relatively shorter MST than mice treated with butanol fraction. The higher burden of parasitaemia may be the reason for the shorter MST. Moreover, their effects in preventing body weight and temperature reduction of mice were not significant compared to their negative control. Due to its better antiplasmodial activity, further analysis was conducted on butanol fraction to isolate active chemical constituents.

Equivalent doses of the crude extract and butanol fraction showed almost a similar percent of parasitaemia inhibition (31.63% vs. 34.58% and 44.55% vs. 45.42%, respectively) and improvement of MST. This may be due to minor effects of the chemical constituents fractionated in aqueous and chloroform fractions, which was supported by the low percentage of yield and less chemosuppression effects of the fractions. On the other hand, the butanol fraction at 200 mg/kg prevented the drop in temperature significantly compared to the negative control unlike the equivalent dose of the crude extract. Generally, both the crude extract and butanol fraction showed similar antiplasmodial activities and are considered active at the tested doses while the aqueous and chloroform fractions can be considered inactive.

Both the isolated compounds also showed chemosuppressive effects in a dose-dependent fashion. The highest (38.19%) suppression was produced by the higher dose of compound C, which is relatively higher than equivalent doses of the crude extract and butanol fraction (38.19% vs. 31.63% and 34.58%). This may be due to less concentration of compound C in the crude and butanol fraction. The result of this study differs from previously reported results which showed that the crude extract demonstrated better antiplasmodial activity than the isolated compounds [25]. Compound C can, therefore, be considered active against rodent malaria. Compound D also reduced parasitaemia level significantly (20.07%). The chemosuppressive effect of compound D was, however, lower than that demonstrated by compound C. This may be due to less schizonticidal activity of the compound. Besides, the chemosuppressive effects of both isolated compounds were not comparable with that of chloroquine phosphate. As in the case of crude extract and butanol fraction, the longest MST of mice treated with both isolated compounds was associated with maximum parasitamia inhibition. In general, compound D is considered inactive at the tested doses.

The effects of the compounds C and D on prevention of body temperature drop in mice on day 4 were not significant in comparison with the negative control. In addition, only compound C significantly prevented body weight loss on day 4 compared to that of the negative control. The reduction of body temperature seen in mice treated with both compounds may be due to their less hyperthermic effects in addition to the effect of parasite burden. Compounds C and D could be one of the aforementioned secondary metabolites in *O. europaea* that have known antiplasmodial activities in other plants. However, they may also be new metabolites not characterized so far.

There are various suggested mechanisms of action for antimalarial activity of different antimalarial compounds including natural products. The most important antimalarial drugs inhibit hemozoin polymerization in the parasite [11, 40]. Some drugs interfere with essential enzymes of the parasite like dihydropteroate synthase and dihydrofolate reductase [41], type II topoisomerase [42], and dihydroorotate dehydrogenase, an enzyme used in the *de novo* pyrimidine synthesis [43]. Other antimalarials affect a special organelle of the *Plasmodium* parasite, called apicoplast, which is involved in protein synthesis [43]. The antiplasmodial mechanism of action of test substances in the present study might, hence, be one of the aforementioned mechanisms or a new mechanism not determined yet.

## 5. Conclusion

The results obtained from the present study revealed that the crude extract, butanol fraction, compound C, and compound D have antiplasmodial activities as evidenced by their ability to suppress *P. berghei* infection in mice in a dose-dependent manner. All the tested samples were also relatively safe. The significant antiplasmodial activity of the plant extract, solvent fractions, and the isolated compounds accompanied with their relative safety may, therefore, confirm the traditional antimalarial claim of *O. europaea*. It can also be suggested that the isolated compounds, especially compound C, could be good candidates for further evaluation for their antimalarial activities. However, in-depth toxicological studies have to be conducted on the safety profiles of the test substances. Furthermore, the structural formula of compounds C and D should be known to determine whether these compounds have good chemical structures so as to be considered good candidates for further investigations.

## Figures and Tables

**Table 1:** Experimental design.

Group	Dose (mg/kg)		
	Crude extract	Fractions	Isolated compounds
Group I	200	100	100
Group II	400	200	200
Group III	600	400	—
Group IV	10 ml/kg^*∗*^	10 ml/kg^*∗*^	10 ml/kg^*∗*^
Group V	25 mg/kg^*∗∗*^	25 mg/kg^*∗∗*^	25 mg/kg^*∗∗*^

^*∗*^Distilled water.^*∗∗*^Chloroquine phosphate. *n* = 6.

**Table 2:** Yield of crude extract and solvent fractions of stem bark of *O. europaea*

Procedure	Original wt. (g)	Yield (g) (%)		
Maceration	1000	221.9 (22.19)		
		Chloroform frac.	Aqueous frac.	Butanol frac.
Fractionation	40	3.10 (7.75)	8.52 (21.30)	26.79 (66.97)

**Table 3:** Effect of the crude extract of *O. europaea* on parasitaemia of *P. berghei* infected mice.

Treatment	Dose (mg/kg)	Antimalarial activities	
		% parasitaemia ± SEM	% suppression
Crude extract	200	21.06 ± 0.64	30.24^a3^
	400	17.32 ± 1.96	42.63^a3^
	600	14.37 ± 0.77	52.40^a3b1^
Dist. water	10 ml/kg	30.19 ± 1.85	0.00
Chloroquine	25	0.00	100.00^abcd3^

Values are expressed as mean ± SEM; *n* = 6; a = compared to negative control; b = compared to 200 mg/kg; c = compared to 400 mg/kg; d = compared to 600 m/kg; 1=*p* < 0.05; 3=*p* < 0.001.

**Table 4:** Effect of the crude extract of *O. europaea* on mean survival time and body weight of *P. berghei* infected mice.

Treatment	Dose (mg/kg)	Body weight (g) ± SEM			MST (day) ± SEM
		D_0_	D_4_	% change	
Crude extract	200	31.34 ± 1.01	29.89 ± 0.98	−4.63^a1*∗*1^	7.83 ± 0.31
	400	33.35 ± 0.61	31.97 ± 0.77	−4.14^a1^	9.83 ± 0.48^a2b1^
	600	33.07 ± 1.24	33.08 ± 1.39	0.03^a3^	10.67 ± 0.49^a3b2^
Dist. water	10 ml/kg	32.97 ± 0.72	28.42 ± 0.23	−13.80^*∗*1^	7.33 ± 0.49
Chloroquine	25	29.62 ± 1.38	31.85 ± 1.05	7.53^a3b2c2d1^	ND

Values are expressed as mean ± SEM; *n* = 6; a = compared to negative control; b = compared to 200 mg/kg; c = to 400 mg/kg; d = compared to 600 mg/kg; 1=*p* < 0.05; 2=*p* < 0.01; 3=*p* < 0.001. ^*∗*^There was significant difference between day 0 and 4 within the group. D_0_ = pretreatment on day zero; D_4_ = posttreatment on day five; ND = no death within the 28-day follow-up.

**Table 5:** Effect of the aqueous, butanol, and chloroform fractions of *O. europaea* on parasitaemia of *P. berghei* infected mice.

Treatment	Dose (mg/kg)	Antimalarial activities	
		% parasitaemia ± SEM	% suppression
Butanol fraction	100	32.86 ± 1.24	26.07^a3^
	200	29.08 ± 0.53	34.58^a3^
	400	24.26 ± 0.17	45.42^a3b2^
Chloroform fraction	100	35.94 ± 1.83	19.15^a1^
	200	34.12 ± 2.66	23.24^a2^
	400	31.81 ± 0.79	28.44^a2^
Aqueous fraction	100	37.50 ± 2.51	15.64
	200	35.68 ± 1.47	19.73^a1^
	400	33.38 ± 1.08	24.90^a2^
Dist. water	10 ml/kg	44.45 ± 2.21	0.00
Chloroquine	25	0.00	100.00^AD3^

Values are expressed as mean ± SEM; *n* = 6; a = compared to negative control; AD = compared to all doses; b = compared to 100 mg/kg of the butanol fraction; 1=*p* < 0.05; 2=*p* < 0.01; 3=*p* < 0.001.

**Table 6:** Effect of the aqueous, butanol, and chloroform fractions of *O. europaea* on mean survival time and body weight of *P. berghei* infected mice.

Treatment	Dose (mg/kg)	Body weight (g) ± SEM			MST (day) ± SEM
		D_0_	D_4_	% change	
Butanol fraction	100	31.67 ± 0.69	30.16 ± 0.68	−4.77^*∗*^2^^	7.50 ± 0.22
	200	31.02 ± 0.61	30.45 ± 0.95	−1.84	8.33 ± 0.49
	400	29.88 ± 0.52	30.08 ± 0.76	0.67^a1^	10.17 ± 0.48^a2b2^
Chloroform fraction	100	25.27 ± 1.26	23.36 ± 1.12	−7.56^*∗*^3^^	7.33 ± 0.33
	200	26.73 ± 1.02	26.11 ± 0.90	−2.32^*∗*^2^^	7.67 ± 0.48
	400	27.83 ± 0.77	26.17 ± 0.55	−5.96^*∗*^1^^	7.83 ± 0.31
Aqueous fraction	100	29.10 ± 0.45	27.93 ± 0.82	−4.02	7.17 ± 0.31
	200	27.67 ± 0.11	26.71 ± 0.57	−3.47	7.67 ± 0.33
	400	27.48 ± 1.13	26.64 ± 1.23	−3.06^*∗*^2^^	8.17 ± 0.48
Dist. water	10 ml/kg	30.15 ± 0.68	27.71 ± 0.97	−8.09^*∗*^2^^	7.17 ± 0.54
Chloroquine	25	28.02 ± 0.27	27.85 ± 0.49	−0.61^a1^	ND

Values are expressed as mean ± SEM; *n* = 6; a = compared to negative control; b = compared to 100 mg/kg of butanol fraction; 1=*p* < 0.05; 2=*p* < 0.01; 3=*p* < 0.001. ^*∗*^There was significant difference between day 0 and 4 within the group. D_0_ = pretreatment on day zero; D_4_ = posttreatment on day five; ND = no death within 28 days.

**Table 7:** Effect of compounds C and D on parasitaemia of *P. berghei* infected mice.

Treatment	Dose (mg/kg)	Antimalarial activities	
		% parasitaemia ± SEM	% suppression
Compound C	100	32.44 ± 0.83	24.82^a3^
	200	26.67 ± 0.69	38.19^a3b2^
Compound D	100	36.77 ± 0.51	14.79
	200	34.49 ± 2.03	20.07^a2^
Dist. water	10 ml/kg	43.15 ± 1.49	0.00
Chloroquine	25	0.00	100^AD3^

Values are expressed as mean ± SEM; *n* = 6; a = compared to negative control; b = compared to 100 mg/kg of compound C; AD = compared to all doses; 2=*p* < 0.01; 3=*p* < 0.001.

**Table 8:** Effect of compounds C and D on body weight and mean survival time of *P. berghei* infected mice.

Treatment	Dose (mg/kg)	Body weight (g) ± SEM			MST (day) ± SEM
		D_0_	D_4_	% change	
Compound C	100	25.40 ± 0.57	23.96 ± 0.59	−5.67^*∗*^1^^	7.89 ± 0.16
	200	25.03 ± 0.52	24.47 ± 0.52	−2.24^a1*∗*1^	8.81 ± 0.19^a3^
Compound D	100	24.47 ± 0.40	22.70 ± 0.70	−7.23^*∗*^1^^	7.25 ± 0.48
	200	24.10 ± 0.66	23.40 ± 0.72	−2.90^*∗*^1^^	8.52 ± 0.45^a1^
Dist. water	10 ml/kg	26.13 ± 0.22	23.65 ± 0.50	−9.49^*∗*^1^^	7.02 ± 0.24
Chloroquine	25	25.53 ± 0.72	26.28 ± 0.75	2.94^ab1^	ND

Values are expressed as mean ± SEM; *n* = 6; a = compared to negative control; b = compared to 100 mg/kg (compound D). ^*∗*^There was significant difference between day 0 and 4 within the group. 1=*p* < 0.05; 3=*p* < 0.001; D_0_ = pretreatment on day zero; D_4_ = post-reatment on day five; ND = no death within 28 days.

## Data Availability

All the data used to support findings of this study are available from the corresponding author when reasonable requests come from concerned bodies.
